# Adaptive Interventions to Promote Change in the 21st Century: The Responsive Feedback Approach

**DOI:** 10.9745/GHSP-D-23-00450

**Published:** 2023-12-18

**Authors:** K. Viswanath, Sohail Agha

**Affiliations:** a Harvard T.H. Chan School of Public Health, Harvard University, Boston, MA, USA.; b Dana Farber Cancer Institute, Boston, MA, USA.; c Behavioral Insights Lab, Seattle, WA, USA.

## Abstract

The RF approach is a framework of intervention development that aims to collect timely data that serve as feedback and provide flexibility, agility, and adaptability to intervention planners and implementers to make changes that enhance their success.

## Key Messages

- Most existing models of intervention designs and implementation lack flexibility for quick adaptation, suffer from time lags between data collection and timely adaptation to course correct, suffer from poor communications between evaluators and implementers within the agency, and lack a forum to have a dialogue among stakeholders to interpret evaluation data and make changes.
- Responsive feedback, an alternative approach to address these limitations, is a framework of intervention development and implementation that is agile, flexible, adaptive, iterative, and actionable.
- Key elements of the responsive feedback approach are that it engages all stakeholders in the design, execution, and review of interventions; improves collaboration between monitoring, learning, and evaluation and implementation personnel; draws on an explicit theory of change; has a built-in data collection system to test assumptions in the theory of change; and has pause-and-reflect sessions to engage all stakeholders on the progress of the intervention, resulting in iterative changes in the intervention toward achieving intervention goals.

## INTRODUCTION

Social change programs or interventions to improve population health through promoting behavior change among individuals or social conditions or infrastructure have been extensively studied over the last few decades. There are several models and frameworks that offer guidance on the processes, methods, and approaches to promoting such interventions.^1^ While proposing a new approach to social change interventions in an earlier article, we raised some questions about existing models of intervention designs and implementation and how they are integrated and executed within an organization.^2^ We argued that most interventions are limited in the following ways.

- Lack flexibility when situations call for quick adaptation to changing and complex social conditions
- Suffer from long-time lags between the availability of data on program performance and the translation of the data to make timely changes
- Have siloes among various stakeholders within the implementation organization (e.g., between monitoring, learning, and evaluation departments and implementers) and between the implementing agency and its users
- Do not have a forum for dialogue among different stakeholders
- Lack a learning mindset and feedback loops, especially where performance metrics are closely tracked to make adjustments in the campaigns

All these reasons could potentially preclude necessary course corrections, leading to potential “failure” of interventions or unintended consequences, such as widening inequalities.^3^

Our characterization is intentionally painted in broad brush strokes, and not all interventions suffer from all these limitations. In fact, most interventions may have some of these elements. These limitations are due to a lack of sound management practices, appropriate designs, and organizational culture, among other reasons.

We highlighted the need for an approach called responsive feedback (RF), a framework of intervention development and implementation that is agile, flexible, adaptive, iterative, and actionable. This approach should reduce uncertainty and allow for decisions to be made based on or at least informed by evidence most of the time. By design and in operation, RF should be conducive to more frequent review of how an intervention is working and enable timely changes that allow continuous iteration and improvement to enhance the quality and impact of an intervention.

We make no claims that RF is totally new. In fact, we argue that the RF approach is part of a family of intervention designs that aim to collect timely data that serve as feedback to intervention planners and implementers to make changes that enhance their success. Among others, this group includes adaptive interventions, feedback loops/mechanisms, rapid cycle innovations, and other frameworks that emerged outside academia. These approaches offer an alternative to conventional approaches and result from increased recognition of the centrality of 4 key features: attention to the context of implementation, focus on the audience or consumers, organizational culture, and timely feedback based on performance data.

## HOW IS THE RF APPROACH DIFFERENT?

Typical interventions or campaigns, as they are usually practiced, particularly when adopted at a larger scale, have often assumed that interventions are fixed and have relied on pre- and post-test designs to assess impact and have maintained a separation between monitoring, learning, and evaluation and implementation personnel. These approaches have been questioned for not accurately reflecting real-world conditions, not being able to explain key ingredients behind successes and failures, and not being able to translate learnings for broader applications. In many cases, this has created challenges for implementing interventions at scale. Experts have proposed the following alternative intervention designs.

- Adaptive intervention designs execute and iterate an intervention based on the needs of individual users and adjust “dosage” for each individual user based on the situation and data and according to certain decision rules.^4^
- Feedback loops/mechanisms are a 2-way stream of communication between the client and the provider of services, as well as between workers and management.^5^
- Rapid cycle innovations encourage internal staff to adopt and adapt practices from outside the organization or within the organization with an emphasis on testing and learning.^6^
- Several frameworks emerged outside academia in the development and health sectors, including the U.S. Agency for International Development's collaborating, learning, and adapting and the Institute for Healthcare Improvement's plan-do-study-act cycles, among others.^7^
- Continuous quality improvement, which emphasizes customer satisfaction among its goals and focuses on underlying processes to achieve those goals, uses data to adapt organizational processes to improve quality and empowers employees to make decisions.

### Elements of RF

The RF approach builds on these alternative intervention design and implementation approaches to offer an integrated, systematic, and systemic method to improve outcomes. It is not a single unified method of intervention as much as a framework with a specific set of elements or activities. Here, we summarize the elements of the RF approach, which are described in more detail at https://the-curve.org.

- It calls for engaging all stakeholders in the design, execution, and review of interventions.
- Though this is debated, RF eschews silos between monitoring, learning, and evaluation and implementation personnel to facilitate seamless communication between them and ensure they are responsive to each other.
- RF argues for inputs from implementers and users on how the intervention is working.
- It has an explicit theory of change that continuously questions and tests assumptions through evaluation data driving the interventions.^8^ The assumptions in such a theory of change could be informed by any number of health behavior theories that could be tested at different levels (e.g., individual, interpersonal, and organizational).^1^
- It recommends using built-in systematic data collection methods to test the assumptions and adapt to changes based on the data.
- RF advocates periodic pause-and-reflect sessions to engage all stakeholders on the progress of the intervention.

These elements, when applied together, offer a framework for dispassionate questioning of intervention assumptions, testing them, and providing timely feedback to the implementers for course corrections (“feedback loops”) to ultimately evaluate the performance of the program. An important element is that sharing of data and course corrections are often done in settings, such as pause-and-reflect sessions, that promote collective ownership in feedback collection and intervention changes. The philosophy of adaptation through timely feedback is built into the process.

In sum, the RF approach is a framework that attempts to codify some of the recent developments in the intervention approaches to provide flexibility and agility to improve the performance of the programs.

However, it is also clear that such an approach may work well in theory but could face multiple challenges in practice. These challenges include donor requirements, the structure and culture of organizations implementing the interventions, concerns about the additional time required to adopt such an approach, resource and operational constraints, and the absence of technical skills and “expertise” needed for such an approach within smaller organizations.^2^ Changes are needed across all these challenges for RF to work effectively. Despite these challenges, both a need and an appeal for an RF approach or something like it have been increasing, and several organizations and development agencies have been adapting versions of it to varying degrees.

## THE RF APPROACH IN PRACTICE

The field of planned social change programs itself is facing questioning for a variety of reasons. As stated earlier, some perceive that the classic or orthodox approach to intervention design and implementation is too hidebound, inflexible, and slow. The introduction of information and communication technologies, particularly mhealth and other variants, has both increased a program's ability to introduce innovations and its potential to collect data rapidly. Yet, structured opportunities to be reflective about the direction of an intervention and to course correct have not necessarily increased. Funding organizations have become much more sensitive to the return on investment and cognizant of their reporting obligations to society. The ecosystem around the intervention that includes different stakeholders, including users and governments, is more sensitive to the intended and unintended consequences of interventions. For these reasons, there is more questioning and demand for new approaches to planned social change programs—a demand that has led to the development of different intervention modalities previously mentioned.

The RF approach is one such answer to the emerging demand for change. A variety of actors, including interventionists, researchers, funders, and development partners, have been using the RF approach in some fashion or another. Although these developments are laudable, no single definition and characterization of RF currently exists, nor is there a place to define, characterize, understand, and apply this approach.

This supplement is a collection of articles that showcase how the RF approach and its iterations work in practice in the interest of codifying what has been learned so far and the next steps of its evolution. The supplement starts with Hornik raising fundamental questions around what are the most useful operational research questions that a program can ask, how does a program determine which operational research questions are worth answering, and what types of answers management is most likely to consider in making changes that correct operational flaws.^9^ The remaining articles in the supplement may be considered as a response to some of these practical considerations through the adoption of the RF approach. Synowiec et al.^10^ and McCloud et al.^11^ provide guidance and arguments on what kind of evaluation designs, data collection strategies, methodological assumptions, and testing strategies are useful depending on the questions to be asked, type and degree of novelty of interventions, and intervention settings. Sharma et al. codeveloped an intervention with adolescents in India.^12^ Ajijola et al.^13^ highlight variations in the use of pause-and-reflect sessions to enhance government leadership in Nigeria to sustain interventions.^13^ Aminu et al.^14^ illustrate how RF may be used to develop and improve performance, leadership, and management in Nigeria. Gillum et al.,^15^ Anieto et al.,^16^ and Trasi et al.^17^ identify variations or sister frameworks of RF adopted for specific interventions in Tanzania, Nigeria, and Burkina Faso, respectively. Okafor et al. present a case promoting demand generation for family planning services,^18^ and Barker et al. describe scaling a gender norms-shifting intervention.^19^ Tomar et al. demonstrate how caregiver engagement in nutrition programs for children can be increased through direct-to-client tools, such as digital coaches.^20^

## CONCLUSIONS

Notably, most of the lead authors or coauthors are practitioners from low- and middle-income countries who have capitalized on the opportunity to share the lessons they have learned while implementing RF interventions. Our hope is that, collectively, these articles paint a comprehensive picture of the RF approach and how it might work in practice in a variety of settings, particularly in low-resource contexts. In addition to showcasing how adaptive interventions work in practice, the variety of case studies, settings, and cultural contexts also serve as models and identify challenges, solutions, and opportunities.
